# What Influences Where They Give Birth? Determinants of Place of Delivery among Women in Rural Ghana

**DOI:** 10.1155/2016/7203980

**Published:** 2016-12-22

**Authors:** Kwamena Sekyi Dickson, Kenneth Setorwu Adde, Hubert Amu

**Affiliations:** Department of Population and Health, University of Cape Coast, Cape Coast, Ghana

## Abstract

*Background*. There is a paucity of empirical literature in Ghana on rural areas and their utilisation of health facilities. The study examined the effects of the sociodemographics of rural women on place of delivery in the country.* Methods*. The paper made use of data from the 2014 Ghana Demographic and Health Survey. Women from rural areas who had given birth within five years prior to the survey were included in the analysis. Descriptive analyses and binary logistic regression were used to analyse the data.* Results*. Wealth, maternal education, ecological zone, getting money for treatment, ethnicity, partner's education, parity, and distance to a health facility were found as the determinants of place of delivery among women in rural Ghana. Women in the richest wealth quintile were three times (OR = 3.04, 95% CI = 0.35–26.4) more likely to deliver at a health facility than the poorest women.* Conclusions*. It behoves the relevant stakeholders including the Ghana Health Service and the Ministry of Health to pay attention to the wealth status, maternal education, ecological zone, ethnicity, partner's education, parity, and distance in their planning regarding delivery care in rural Ghana.

## 1. Introduction 

Although advancements have been made in medical care and public health, about 830 women died every day as a result of complications related to pregnancy and childbirth in 2015 [1]. Of this, 180 occurred in Southern Asia and 550 in sub-Saharan Africa, compared to just five in developed countries. Maternal mortality is thus considered the leading cause of death among females aged 15–49 years worldwide and particularly in developing countries [2, 3]. World Health Organisation, for instance, noted that 99% of all maternal deaths occur in developing countries [1].

Most of these deaths are concentrated around the time of delivery [4] and their major causes are obstructed labour, sepsis, haemorrhage, and hypertensive disorders [5]. Lwelamira and Safari [6] opined that most of the recorded maternal deaths were avoidable if timely skilled delivery services were provided to the women. Moreover, readily accessible and appropriate care in case of complications and effective postnatal care within the first 24 hours of delivery are strategies that can be put in place to reduce the high maternal deaths recorded in developing countries [7]. However, a significant proportion of women in developing countries does not have the opportunity of giving birth with skilled assistance and at health facilities [8]. Utilisation of health facilities is influenced by several factors including availability, distance, cost, quality, socioeconomic factors, and personal health beliefs [9].

Wide disparities usually exist between urban and rural areas in the utilisation of health facilities, with rural areas being the disadvantaged [1]. Alemayehu and Mekonnen [10] in an Ethiopian study, for instance, found that health facility delivery was 7.15 times more likely to be used by urban residents compared to residents in rural areas. When other confounding sociodemographic variables were controlled, the adjusted odds of health facility use at delivery were 5.46 times higher among urban residents compared to the rural residents.

In Ghana, the distribution of health care facilities is skewed towards urban areas [11]. Over 65% of medical doctors in the country are concentrated in two urban-based teaching hospitals: Komfo Anokye in Kumasi and Korle Bu in Accra [11]. In the Ashanti Region, one of ten regions in the country, medical doctors in the Kumasi Metropolis alone constitute over 85% of all medical doctors in that region [11]. The medical doctors mainly refuse posting to rural areas, even with extra remuneration packages. and this is due to poor environmental conditions and inadequate social infrastructure in the rural areas [12]. The skewed distribution pattern of health facilities in favour of urban areas also reflects their utilisation rate between the rural and urban areas [11]. This condition is expedited by the fact that distance is an important determinant of health facility use [11, 13, 14]. Buor [15] also conducted a comparative analysis of health services utilisation in rural and urban areas in Ghana and identified the major determinants of health service utilisation in urban areas as distance, service cost, health status, and quality of service. In rural areas, the author also identified distance, service cost, education, and income [15].

The 2014 Ghana Demographic and Health Survey shows that regarding service utilisation rates, even though there have been increases in antenatal and postnatal care, as well as delivery care, disparities still exist between rural and urban areas with the rural areas being the disadvantaged [16]. Concerning antenatal care, for instance, even though it is at a universal coverage, the percentage of women receiving antenatal care from a skilled provider stood at 98.6% for urban areas while that of rural areas was 96.2% [16]. With delivery care, while 90.2% of women in urban Ghana delivered in a health facility with just 9.4% doing so at home and the rest 0.4% at “other” places, in rural Ghana 59% delivered in health facilities while 40.6% did so at home and 0.4% at “other” places [16]. With postnatal care, in urban Ghana, 90.2% of women had a postnatal checkup in the first two days after birth. In rural Ghana, however, 73.9% of women who gave birth went for a postnatal checkup in the first two days after birth [16]. It was also realised that while women with no postnatal checkup constituted 7% in urban Ghana, 21.4% of women in rural Ghana did not have any postnatal checkup after birth [16].

Despite the fact that rural areas lag behind urban areas in terms of health facility use which has implications for maternal mortality in those areas, there appears to be a paucity of empirical literature in Ghana with focus on rural areas and their utilisation of health facilities. This paper, therefore, examines the relationship between the sociodemographic background of women and their choices of the place of delivery. The study contributes to the existing literature on skilled delivery among women in reproductive age in the country by outlining the sociodemographic predictors of choice of place of delivery in the country.

## 2. Material and Methods

### 2.1. Setting

The republic of Ghana is centrally located on the West African Coast and has a total land area of 238,533 square kilometers. Ghana is a low-lying country except for a series of hills on the eastern border and Mountain Afadjato, the maximum point (883 metres) above sea level which is west of Volta Region. Ghana can be divided into three ecological zones, namely, Savannah zone, Forest Zone, and the Sandy Coastline backed by coastal plains (coastal zone). The dominant ethnic groups in the country are Akan (47.5%), Mole-Dagbani (16.6%), Ewe (13.9%), Ga–Dangme (7.4%), Gurma (5.7%), Guan (3.7%), Grusi (2.5%), other (1.4%), and Mande (1.1%) [17]. Ghana has about 51 percent of its population in urban areas and 49 percent in rural areas. There are 3217 functional health facilities, out of which 4 are teaching hospitals. There are also 9 regional hospitals, 3 psychiatric hospitals, 11 polyclinics, 59 Christian Health Association of Ghana (CHAG) hospitals, 10 Islamic hospitals, 96 government hospitals, 156 private hospitals, and 22 quasi-government hospitals. The majority of these health facilities are found in the urban areas [17].

### 2.2. Source of Data

The study made use of secondary data from the 2014 Ghana Demographic and Health Survey (GDHS). The GDHS is a nationwide survey which is conducted by the Ghana Statistical Service and the Ghana Health Service with technical support from ICF International through the MEASURE DHS program. The GDHS focuses on child and maternal health and is designed to provide adequate data to monitor the population and health situation in the country. GDHS gathers information on various demographic and health variables including maternal care, fertility, contraception, child health, family planning, HIV and AIDS, nutrition, and malaria. A total of 9,396 women, ages 15–49, from 12,831 households covering 427 clusters throughout Ghana were interviewed. The survey had a response rate of 97%. Our study, however, focused on women in rural areas: a sample of 2,278. Since preexisting data was used, ethical clearance was not required. Permission to use the data was obtained from MEASURE DHS after assessment of the proposal. The dataset is available at http://www.measuredhs.com.

The study made use of secondary data from Ghana Demographic and Health Survey (GDHS) which has a standard questionnaire. Ethnicity was based on the types of ethnic groups in Ghana and the distance to a health facility was based on whether the respondent considered the distance from her home to the health facility as a problem. All the variables were based on the standard questionnaires of GDHS.

In the 2014 GDHS report, information on place of delivery was only presented descriptively on the rural-urban dichotomy. Our study, therefore, provides extra information on place of delivery in the country as an added value of further analysis of the GDHS by focusing on only rural areas which were found in the survey to have been disadvantaged in terms of health facility delivery. Our study also provides statistical information on the factors which influence the place of delivery among rural women in the country.

### 2.3. Analysis

The outcome variable employed for this study was place of delivery. It was coded 0 = “home” and 1 = “health facility” since it was a dichotomous variable. A discrete choice model was employed to show how the explanatory variables correlated with the outcome variable. Specifically, the binary logistic regression was employed since it allows the predictions on a mixture of continuous and categorical variables.

Nine explanatory variables were used: maternal age, wealth index, educational level, ecological zone, getting money for treatment, ethnicity, parity (birth order), partner's education, and distance to a health facility. Ecological zone was created by collapsing the ten regions into three: Central, Western, Greater Accra, and Volta as the Coastal zone; Ashanti, Eastern, and Brong-Ahafo were classified as the Forest zone, while the Upper East, Northern, and Upper West regions were grouped as the Savannah zone. The Coastal, Forest, and Savannah zones are established ecological zones in Ghana.

Parity (birth order) was categorised as one birth, two births, three births, and four births or more. Maternal age was categorized into 15–19, 20–24, 25–29, 30–34, 35–39, 40–44, and 45–49. Education was classified into four categories: no education, primary education, secondary education, and higher education. Distance to a health facility was categorised as big problem and not a big problem. Ethnicity was grouped as Akan, Ga/Dangme, Ewe, Guan, Mole–Dagbani, Grusi, Gruma, Mande, and other. Partner's education was classified into four categories: no education, primary education, secondary education, and higher education. The wealth index was grouped as poorest, poorer, middle, richer, and richest. Getting money for treatment was captured as big problem and not a big problem.

The explanatory variables used in the analysis were purposively selected based on previous literature. Chi-square test was done in Table 1 to check if they were significantly associated with the outcome variable; those variables that were significantly related were then used in the multivariate regression analysis. All results were weighted to offset the biases of under and oversampling as well as reporting associated with national surveys. All analyses were conducted using STATA, version 13.

## 3. Results and Discussion

### 3.1. Results

Table 1 presents the places of delivery based on sociodemographic characteristics. Eight of the explanatory variables showed significant univariate associations with the dependent variable: wealth index, maternal education, ecological zone, getting money for treatment, ethnicity, partner's education, parity, and distance to a health facility. It was observed that women aged 45–49 years had the highest proportion of home delivery (51.2%). While the poorest had a 46.3% proportion of health facility delivery, the richest had 91.6%.

The choice of health facility for delivery was found to have increased by the level of education attained by the women and their partners. Regarding ecological zone, women from the savannah zone had the highest proportion of home deliveries (52.7%) compared to women from the forest zone who had the highest proportion of health facility delivery (65.6%). Women who had a big problem getting the money needed for treatment (46%), Gurmas (73.9%), women with four births or more (46.1%), and those who saw distance to a health facility as a big problem (51.8%) had the highest proportions of births delivered at home.

Table 2 presents results of the binary logistic regression conducted for place of delivery and the explanatory variables. The results show significant unique contributions of education (primary, secondary, and higher), wealth status (middle, richer), parity (four births or more), partner's education (primary, secondary, and higher), distance to health facility (not a big problem), ecological zone (forest, savannah), and ethnicity (Mole–Dagbani, Grusi, and Gurma) on place of delivery. Women in the richer (OR = 4.72, 95% CI = 2.42–9.21) and richest (OR = 3.28, 95% CI = 0.38–28.27) wealth quintiles were four and three times, respectively, more likely to deliver at a health facility than women in the poorest wealth quintile. Those with two births (OR = 0.82, 95% CI = 0.57–1.16), three births (OR = 0.79, 95% CI = 0.56–1.12), and four birth or more (OR = 0.66, 95% CI = 0.49–0.90) were all less likely to deliver in a health facility compared with those with one birth. It was also realised that the higher the level of education of partner, the more likely it was for women to deliver in a health facility. Women in the forest (OR = 1.34, 95% CI = 1.04–1.72) and savannah (OR = 1.37, 95% CI = 0.95–1.97) zones were also more likely to deliver in a health facility than those in the coastal zone. Women who did not consider distance to health facilities as a big problem were more likely to deliver in a health facility compared with those who considered distance as a big problem (OR = 1.56, 95% CI = 1.27–1.93).

## 4. Discussion

Childbearing is an important event in the life of most women and is positively associated with the ultimate goals of happiness, completeness, and family integration [18]. The place of delivery chosen by women is therefore very important with health facility being the most ideal [19]. Our study indicates that various factors impact the choice of women regarding the place of delivery, some of which are out of their control.

Our findings show that wealth plays an important role in the choice of place of delivery among women in rural Ghana. We realised, for instance, that health facility delivery mainly favoured the rich in both the descriptive and the inferential analyses conducted. For instance, the richest women were three times more likely to deliver at a health facility than the poorest ones. This finding is in line with previous findings regarding the choice of place of delivery and the disparity between poor and rich women with regard to health facility delivery [5, 20–22].

The findings where the poorest women had the worst utilisation of health care facilities for delivery were probably because they have to pay for health care, which is usually problematic for them as argued by Amu and Dickson [23]. The authors noted that even though Ghana's National Health Insurance Scheme, introduced in 2003, was designed to be pro-poor, many of the poor are unable to subscribe to the scheme due to their inability to afford annual premiums required to access health care free of all charges at the point of service delivery.

The role of wealth in predicting place of delivery among rural women is also corroborated by our findings which showed that women who said getting the money needed for treatment was a big problem were less likely to deliver at a health facility than those who said getting the money needed for treatment was not a big problem.

We found that rural women in the coastal zone had the least probability of delivering in a health facility while those in the savannah zone had the highest probability. Our findings, therefore, debunk assertions that utilisation of health care services including delivery care is more prevalent in Coastal Ghana than in Savannah Ghana [24]. This finding may be due to the differential access and experiences with higher standards of care, largely in the private sector as experienced in the coastal zone [25], while being less likely to be experienced in any of the regions constituting the savannah zone [26].

The findings may also be due to the fact that as women in the coastal zone usually experience the multiplicity of health facilities, their perception of the quality of maternal health care services provided to them becomes negative due to negative attitude of staff towards them, shortage of qualified staff, and a lack of essential logistics such as drugs and resuscitation materials necessary for emergency obstetric care as argued in previews studies [3, 19, 27].

Our results also point to the significant role played by education in the choices women make regarding the place of delivery. Thus, it was realised that the probability of giving birth at a health facility increased by education, not only for the women but also for their partners. These findings are thus in congruence with previous studies which found education as a strong determinant of health care service and facility use [23, 28–30]. The fact that partner's education predicted the choice of place of delivery among the women, however, supports the argument that men usually control decision-making in the home, including women's health decision-making [31, 32].

Women who considered distance to a health facility as a big problem were found to have been less likely to actually deliver at a health facility compared with those who did not consider distance as a big problem. This points to long distances that are usually travelled by rural women in order to access maternal health care as found in previous studies [23, 27]. Roads in these rural areas are also mainly in a deplorable state [33, 34]. As such, women who could not afford the cost of transportation which is expensive due to the bad nature of the road [35] had no option but to deliver at home as realised in previous studies [36].

Our findings which showed that women with one birth were more likely to utilise health facilities at delivery than those with two or more births confirm Umurungi's [37] argument that women with one birth are usually more likely to deliver their next births at a health facility. This may be due to the fact that primiparous women are considered to be naturally at a higher risk of maternal complications than multiparous women [38]. As such, they access health facility delivery more than multiparous women [35]. The high proportion of home deliveries realised among multiparous women may be due to their preference for Traditional Birth Attendants (TBAs) as posited by Ntambue et al. [38]. The authors argued that TBAs were preferred because they are considered as being more caring and friendly and also provide complementary services such as food and bathing water to the women when they give birth to them compared to their health facility deliveries.

Despite the significant findings made in our study, the limitation of the data used is worth mentioning. Thus, by relying on a cross-sectional data, it is impossible to account for unobserved heterogeneity. Moreover, associations found between the dependent and the explanatory variables may vary over time.

## 5. Conclusions

We found that wealth index, maternal education, ecological zone, getting money for treatment, ethnicity, partner's education, parity, and distance to a health facility are the determinants of place of delivery among women in rural Ghana. It thus behoves the relevant stakeholders including the Ghana Health Service and the Ministry of Health to pay attention to these demographics in their decisions regarding delivery care in rural Ghana, which includes citing of health facilities and public education on the need for health facility delivery. Specifically, considerations should be given to the poor, multiparous women, those with no or low levels of education, and coastal dwellers (Volta, Greater Accra, Central and Western regions).

## Figures and Tables

**Table 1:** Background characteristics and place of delivery.

Variable	Place of delivery		*P* value
	Home	Facility	
	*N* = 906 (100%)	*N* = 1372 (100%)	
*Maternal age*			0.107
15–19	23 (38.0%)	38 (62.0%)	
20–24	153 (40.0%)	228 (60.0%)	
25–29	206 (36.4%)	360 (63.6%)	
30–34	223 (45.5%)	267 (54.5%)	
35–39	165 (38.4%)	288 (63.6%)	
40–44	93 (38.6%)	148 (61.4%)	
45–49	45 (51.2%)	43 (48.8%)	
*Wealth index*			0.000
Poorest	452 (53.7%)	389 (46.3%)	
Poorer	301 (41.9%)	418 (58.1%)	
Middle	138 (29.7%)	327 (70.3%)	
Richer	13 (5.9%)	207 (94.1%)	
Richest	3 (8.4%)	31 (91.6%)	
*Educational level*			0.000
No education	468 (53.4%)	409 (46.6%)	
Primary	213 (41.1%)	305 (58.9%)	
Secondary	223 (26.4%)	620 (73.6%)	
Higher	3 (6.9%)	38 (93.1%)	
*Ecological zone *			0.000
Coastal zone	302 (34.7%)	569 (65.3%)	
Forest zone	256 (34.4%)	490 (65.6%)	
Savannah zone	348 (52.7%)	313 (47.3%)	
*Getting money needed for treatment*			0.000
Big problem	531 (46.0%)	623 (54.0%)	
Not a big problem	375 (33.4%)	749 (66.6%)	
*Ethnicity *			0.000
Akan	296 (31.0%)	659 (69.0%)	
Ga/Dangme	38 (43.0%)	52 (57.0%)	
Ewe	104 (37.6%)	172 (62.4%)	
Guan	16 (34.3%)	30 (65.7%)	
Mole–Dagbani	181 (37.8%)	297 (62.2%)	
Grusi	28 (37.9%)	46 (62.1%)	
Gurma	217 (73.9%)	77 (26.1%)	
Mande	5 (15.9%)	27 (84.1%)	
Other	21 (66.8%)	10 (33.2%)	
*Parity *			0.000
One birth	90 (27.5%)	238 (72.5%)	
Two births	139 (36.6%)	240 (63.4%)	
Three births	146 (34.9%)	273 (65.1%)	
Four births or more	531 (46.1%)	621 (53.9%)	
*Partner's education *			0.000
No education	396 (56.0%)	311 (44.0%)	
Primary	129 (40.9%)	187 (59.1%)	
Secondary	361 (32.0%)	767 (68.0%)	
Higher	20 (15.8%)	106 (84.3%)	
*Distance to health facility*			0.000
Big problem	422 (51.8%)	392 (48.2%)	
Not a big problem	485 (33.1%)	979 (66.9%)	

**Table 2:** Binary logistic regression on place of delivery among women in Ghana.

Independent variable	Odds ratio	95% confidence interval
*Educational level*		
No education	Ref	Ref
Primary	1.30^*∗∗*^	1.01–1.67
Secondary	1.84^*∗∗∗*^	1.40–2.42
Higher	5.94^*∗*^	0.73–48.06
*Wealth index*		
Poorest	Ref	Ref
Poorer	1.16	0.90–1.49
Middle	1.60^*∗∗*^	1.14–2.23
Richer	4.72^*∗∗∗*^	2.42–9.21
Richest	3.28	0.38–28.27
*Parity *		
One birth	Ref	Ref
Two births	0.82	0.57–1.16
Three births	0.79	0.56–1.12
Four births or more	0.66^*∗∗∗*^	0.49–0.90
*Partner's education *		
No education	Ref	Ref
Primary	1.50^*∗∗*^	1.13–2.01
Secondary	1.54^*∗∗*^	1.18–2.01
Higher	2.14^*∗∗*^	1.15–3.98
*Distance to health facility*		
Big problem	Ref	Ref
Not a big problem	1.56^*∗∗∗*^	1.27–1.93
*Getting money need for treatment*		
Big problem	Ref	Ref
Not a big problem	1.00	0.80–1.23
*Ecological zone *		
Coastal zone	Ref	Ref
Forest zone	1.34^*∗∗*^	1.04–1.72
Savannah zone	1.37^*∗*^	0.95–1.97
*Ethnicity *		
Akan	Ref	Ref
Ga/Dangme	0.80	0.47–1.34
Ewe	1.03	0.74–1.43
Guan	1.37	0.73–2.59
Mole–Dagbani	1.79^*∗∗*^	1.23–2.59
Grusi	1.47	0.89–2.40
Gurma	0.47^*∗∗*^	0.31–0.71
Mande	3.15^*∗∗*^	1.33–7.46
Other	0.44^*∗*^	0.19–1.03

Ref = Reference. ^*∗*^*P* < 0.05;  ^*∗∗*^*P* < 0.01;  ^*∗∗∗*^*P* < 0.001.
